# Student advanced trauma management and skills (SATMAS): a validation study

**DOI:** 10.1007/s00068-024-02456-4

**Published:** 2024-02-02

**Authors:** Prakrit R. Kumar, Jamie Large, Nagarjun Konda, Yousuf Hashmi, Oluwasemilore Adebayo, Meenakshi Sivaraman, Justine J. Lee

**Affiliations:** 1 Lister Hospital, East and North Hertfordshire NHS Trust, Stevenage, Hertfordshire SG1 4AB UK; 2 Kingston Hospital NHS Foundation Trust, London, UK; 3 University Hospital Coventry & Warwickshire, Coventry, UK; 4 University of Warwick, Coventry, UK; 5 College of Medical and Dental Sciences, University of Birmingham, Birmingham, UK; 6 Rock Bridge High School, Columbia, MO USA; 7 Major Trauma Service, Queen Elizabeth Hospital, Birmingham, UK

**Keywords:** Undergraduate, Medical education, Trauma and orthopaedics, National evaluation

## Abstract

**Introduction:**

Despite trauma accounting 9% of global mortality, it has been demonstrated that undergraduate trauma teaching is inadequate nationally and worldwide. With COVID-19 exacerbating this situation, a scalable, accessible, and cost-effective undergraduate trauma teaching is required.

**Methods:**

Our Continual Professional Development United Kingdom (CPUDK)-accredited University Hospitals Birmingham (UHB) Major Trauma Service (MTS) affiliated programme consisted of seven biweekly pre-recorded sessions that were delivered online through the Moodle educational platform to University of Birmingham students. Pre- and post-randomised session-specific multiple-choice questions (MCQs) and anonymous feedback forms were administered.

**Results:**

There were 489 student responses, with 63 students completing all seven sessions. On an 8-point scale, students’ objective knowledge scores increased by a mean of 1.2 (*p* < 0.001). Using a 5-point Likert scale, students also showed improvement in subjective outcomes including their confidence in assessing trauma patient (absolute difference (AD) 1.38, *p* < 0.001), advising initial investigations and formulating initial management plans (AD 1.78, *p* < 0.001) and thereby their confidence to manage a trauma patient overall (AD 1.98, *p* < 0.001). A total of 410 student responses endorsed the online delivery of SATMAS through Moodle and recommended SATMAS to future medical students.

**Conclusion:**

SATMAS has demonstrated positive student feedback and extensive recruitment from only one centre, demonstrating that our programme can be an indispensable low-cost learning resource that prepares undergraduate medical students for their trauma exams and informs the implementation of clinical skills required by all doctors. We publish our pilot study findings to encourage similar teaching programmes to be adopted at other universities nationally and internationally, to synergistically benefit students, tutors, and ultimately patients, on a larger scale.

**Supplementary Information:**

The online version contains supplementary material available at 10.1007/s00068-024-02456-4.

## Introduction

Trauma is responsible for 9% of global mortality and is the leading cause of death in the under 45s in the UK [1, 2]. Junior doctors are expected to conduct the initial assessment and management of a large proportion of trauma patients, with consultants absent at 30% of trauma calls in the UK [3]. Despite this, acute trauma care is an area of practice in which many junior doctors report feeling underprepared, with one main reason for this being a lack of formal undergraduate teaching [4, 5]. Studies over the previous decade have identified a lack of undergraduate trauma teaching in both the UK and globally [6, 7]. In 2015, the General Medical Council (GMC) set broad learning outcomes (LOs), stating that medical schools must prepare students to manage medical emergencies but made no specific recommendations for trauma [8], with medical schools acting as autonomous organisations able to decide which aspects of trauma to teach. Inadvertently, this approach may create disparities in teaching practice and exposure to procedural skills between institutions. Indeed, in 2022, our study (National Evaluation of Trauma Teaching for Students (NETTS)) of medical graduates from 39 UK medical schools identified ongoing deficiencies, with only 34.7% of participants reporting that trauma teaching was sufficient [9]. COVID-19 had aggravated this already non-optimum system, with clinical placements being cancelled and thereby in-person teaching opportunities being impacted [10, 11]. However, the expectations of junior doctors remain the same, irrespective of medical school or location. Thus, in line with our previous NETTS study, most graduates feel that a common nationally standardised course in trauma would be a useful adjunct to teaching at medical school (93.7%) to meet common competencies of foundation doctors.

Medical schools use observed structured clinical examinations (OSCEs) to assess the quality of clinical trauma procedural skills. With significant restrictions in staffing quotas, financial restraints, and national guidance, there is reduced undergraduate-tailored clinical learning opportunities for students to practice clinical skills necessary to achieve fluency to pass OSCEs. Where previous online courses in other specialities have shown to improve confidence in passing OSCEs and clinical skills [12], we aim to assess the potential of undergraduate-tailored trauma teaching programme in improving students’ confidence in passing their trauma and perioperative exams, and pave way for further research for its development in undergraduate medical curricula.

With insufficient undergraduate trauma teaching in the UK and abroad, there is a need for scalable and cost-effective trauma teaching which can be implemented in medical schools worldwide. Once established, eLearning is less dependent on tutor availability, reduces both travel costs and time spent away from the workplace, and can have relatively low maintenance costs [13]. Moreover, eLearning is flexible for learners and can be accessed regardless of location. COVID-19 has resulted in mainstream shift towards online medical education [14], with positive student satisfaction and improvements in long-term knowledge shown from eLearning in other specialities [15–17]. Although the restrictions of COVID-19 may no longer be present, the eLearning structures and systems implemented during the pandemic served as a learning tool and example in our initiatives to create this eLearning resource. Despite this huge success of online learning [18, 19], there is limited literature on efficacy of online undergraduate trauma medical education, demonstrating its own unique challenges. Therefore, we devised a low-cost undergraduate eLearning **s**tudent **a**dvanced **t**rauma **m**anagement **a**nd **s**kills (SATMAS) programme and piloted this programme at a UK university. Through the delivery of SATMAS, we aim to assess the effectiveness and determine feasibility of an online education platform and programme in trauma for undergraduates, and laying the foundation for future research.

The aim of this study were threefold: (1) deliver standardised, high-quality teaching to improve the confidence of UK undergraduates preparing for their trauma and perioperative exams, (2) develop medical students’ confidence in knowledge and skills for common challenges as future doctors, and (3) determine suitability of pre-recorded online delivery of undergraduate trauma programme.

## Methods

SATMAS, designed by the University of Birmingham (UoB) Trauma and Orthopaedics (T&O) Society in collaboration with Queen Elizabeth Hospital Birmingham Major Trauma Service, was created to prepare all medical students for their upcoming OSCE and future placements as foundation year doctors. The teaching team, consisting of three UoB T&O society committee members, a foundation doctor digital teaching fellow and Major Trauma Service (MTS) Educational Programme Director (EDP), produced all assessment materials and were responsible for coordinating with the tutors in preparation for each session.

## Study design

### Course

SATMAS consisted of seven biweekly CPD UK-accredited pre-recorded 60-min sessions from October 2020 to February 2021. The course was designed such that students were required to complete each session within three weeks of opening and would not be able to progress without completing the previous session, to provide structured timetable (Appendix 1).

### Content development

The learning outcomes were designed to cover the areas of trauma-related knowledge and skills that a junior doctor would be expected to understand and perform as part of a trauma team, based on university and GMC undergraduate curricula. The content was created to ensure that sessions were tailored for medical students, with an OSCE-focus [20]. This content was approved by a MTS EDP for appropriateness. Tutors then used this content to design and deliver presentations. Demonstration and teaching of appropriate clinical skills were pre-recorded by trauma trainees in clinical teaching rooms, emulating OSCE-style environment. Finally, foundation doctors reviewed presentations and skills videos to ensure all content was conveyed, offering four quality checks before delivery (Appendix 2), akin to previous literature [12].

### Delivery

Each session, using the pre-recorded Moodle platform, followed the same structured approach (Appendix 3) to ensure that students were able to cover all aspects of the topic, learn OSCE-style pre-recorded demonstrations of clinical skills, and apply their knowledge to interactive case-based-discussions (CBD) in limited time.

Prior to piloting the teaching programme, an alpha test was conducted amongst nine members of the UoB T&O Society who had no prior involvement in the development of the programme. Any identified technical problems were resolved prior to the main pilot. The course was then delivered during the first semester of the academic year.

### Recruitment

#### Medical students

Students from the UoB were recruited via word of mouth, social media, and local medical school mailing list. Students were required to sign up using a Google form and were then given a Moodle account and access to the course. Enrolment on the course was voluntary and free of charge.

#### Tutors

Trauma-trainees and consultants in the MTS with an interest in medical education and up-to-date clinical experience were recruited to deliver sessions.

### Outcome measures: feedback forms and MCQ questions

#### MCQs

Before and after each teaching session, an eight-question case-based session-specific test was delivered to students to assess improvements in knowledge. Previous literature that used only four MCQ test scores was discredited and the optimum number of MCQs was not defined in the literature [12, 21–23]. In this study, we used eight MCQs given the depth of each topic. Single best answer (SBA) multiple choice questions as adopted by many universities were utilised. For each session, a bank of questions was created by foundation doctor in acute care and reviewed by teaching committee and MTS Educational Programme Director, from which the test questions for each session were randomly drawn. Randomisation and independent review by a specialist in major trauma served to reduce potential bias and ensure equalisation of question difficulty across pre-session and post-session tests.

#### Feedback forms

Pre- and post-session feedback forms assessed students’ confidence in the specific areas of knowledge and skills pertaining to each session and students’ satisfaction with session delivery and tutors (Appendix 4A, 4B). The pre- and post-course feedback form was administered to assess improvement in students’ confidence in learning outcomes from programme and students’ satisfaction with the course (Appendix 4C, 4D).

### Statistical analysis

All data from feedback forms was collected and collated on an Excel Spreadsheet and analysed using SPSS Statistics (IBM, SPSS, V25). The Wilcoxon matched pairs signed rank test was used to compare MCQ scores, confidence prior and post teaching session, and prior and post-course, with *p* < 0.05*, *p* < 0.01**, and *p* < 0.001*** considered statistically significant. Results are presented as absolute difference, using GraphPad Prism 5 software (GraphPad, Sand Diego, CA, USA). Subgroup analysis of the Likert scale scores before and after the teaching sessions was performed according to each teaching session via one-way analysis of variance and post hoc Bonferroni test.

### Ethics

This study was registered and approved by the University Hospital Birmingham Clinical Audit and Registries Managements Service. The project protocol was processed and registered through the NHS Health Research Authority [24], which determined that this study did not require NHS Research Ethics approval (Appendix 5), similar to other participant evaluations [25–28].

## Results

**Fig. 1:**
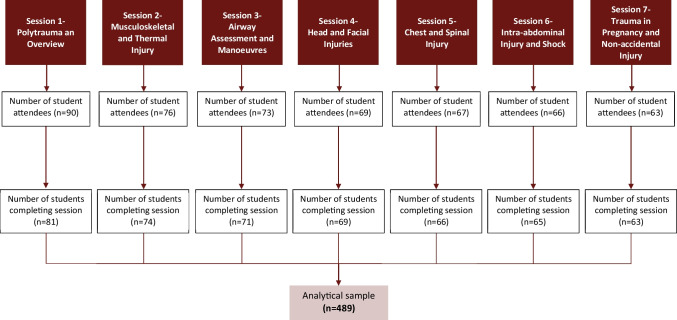
Flowchart of the number of participants in each stage of the programme

Amongst students who started the course, 93% were in 3rd–5th year of medical school. SATMAS recorded 489 student responses across the seven sessions, with 65 students completing the whole teaching programme (Fig. 1). Prior to the teaching programme, of the 65 students, 58.1% were dissatisfied with medical school curricula for trauma teaching, with 88.9% having had less than 5 h of trauma teaching throughout their medical school education. Further, out of 65 students, 60.3% spent less than 5 h in A&E and 85.7% had limited experience of trauma-themed simulation sessions as part of their degree. SATMAS delivered training to address these inadequacies.

### Objective 1—To deliver standardised, high-quality teaching to improve the confidence of UK undergraduates in trauma and their trauma/perioperative exams

**Fig. 2:**
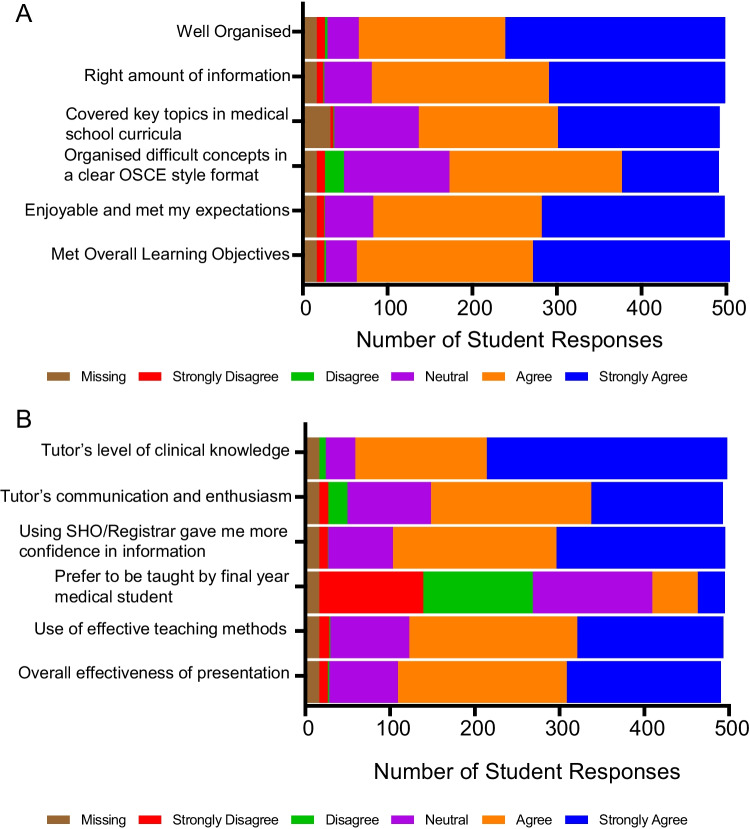
SATMAS conveyed content well (*n* = 489). Based on a five-point Likert scale, where 1 is strongly disagree and 5 is strongly agree; 489 student responses demonstrating their Likert score of agreement to **A** appropriateness of content for undergraduates and **B** effectiveness of online tutors to deliver SATMAS

Most students reported that sessions were well organised (87.7%), had right amount of information (83.8%), covered key topics in medical school curricula (75.1%), organised difficult concepts in a clear OSCE style format (66.3%), was enjoyable and satisfied expectations (85.7%), and met stated LOs (87.7%) (Fig. 2A). Students also agreed that each of the session’s LOs was met (Table 1).

**Table 1:** Student satisfaction that learning outcomes were met, stratified by session

Learning objective	Confidence				Students that supported the learning outcome were met, *n* (%)
	Before	After	Improvement	*p*-value	
Session 1: polytrauma (*N* = 81)					
Overall knowledge of polytrauma	1.71	3.68	1.97	< 0.0001	53 (65.4)
Initial investigations and initial management plan for polytrauma patient	1.90	3.68	1.78	< 0.0001	54 (66.7)
Interpretation of orthopaedic x-rays	2.02	3.68	1.66	< 0.0001	53 (65.4)
Session 2: musculoskeletal Injury and burns (*N* = 74)					
Overall knowledge of musculoskeletal injury and burns	2.23	3.77	1.54	< 0.0001	51 (69.0)
Assessment of patient with burns	1.76	3.72	1.96	< 0.0001	48 (64.9)
Assessment of patient with traumatic musculoskeletal injury	2.15	3.81	1.66	< 0.0001	52 (70.3)
Session 3: airways (*N* = 71)					
Overall knowledge of airways assessment	2.80	4.14	1.34	< 0.0001	59 (83.1)
Assessment of airway of an unconscious trauma patient	3.79	4.04	1.25	< 0.0001	54 (76.1)
Session 4: head and facial injury (*N* = 69)					
Overall knowledge of head and facial injuries	1.97	3.91	1.94	< 0.0001	49 (71.0)
Assessment of patient with a head injury	1.83	3.74	1.91	< 0.0001	41 (59.4)
Assessment of patient with a facial injury	1.72	3.71	1.99	< 0.0001	41 (59.4)
Assessment of patient with epistasis	2.28	4.04	1.77	< 0.0001	52 (75.4)
Assessment of state of consciousness of patient using GCS scoring system	2.74	4.19	1.45	< 0.0001	55 (79.7)
Session 5: chest and spinal injury (*N* = 66)					
Overall knowledge of chest and spinal injuries	2.11	3.71	1.60	< 0.0001	41 (62.1)
Assessment of patient with chest injury	2.08	3.70	1.62	< 0.0001	43 (65.2)
Assessment of patient with spinal injury	1.97	3.60	1.63	< 0.0001	39 (59.1)
Session 6: intra-abdominal injury and shock (*N* = 65)					
Overall knowledge of abdominal trauma and shock	2.26	3.98	1.72	< 0.0001	50 (76.9)
Assessment of patient with abdominal injury	2.06	3.82	1.76	< 0.0001	45 (69.2)
Assessment of patient with shock	2.39	3.85	1.46	< 0.0001	47 (72.3)
Session 7: pregnant patient with major trauma and non-accidental injury (*N* = 63)					
Overall knowledge of pregnant trauma patient and non-accidental injury	1.61	3.88	2.27	< 0.0001	42 (66.7)
Assessment of patient with suspected non-accidental injury	1.88	3.72	1.84	< 0.0001	38 (60.3)
Assessment of pregnant patient with major trauma	1.43	3.58	2.15	< 0.0001	35 (55.6)

The majority of students agreed on the overall effectiveness of our teaching format (80.2%) and the delivery of content by our online tutors (77.7%) (Fig. 2B). There was an improvement in knowledge from pre- to post-session, with a mean improvement of 1.19 (*p* < 0.0001) (Table 2) on an 8-point scale. As a result, students who completed the whole course were more confident in encountering a trauma-based scenario in a future OSCE, as determined using a 5-point Linkert scale (AD 1.93, *p* < 0.0001).

**Table 2:** Student test scores stratified by session

Session	*N*				
		Test score			
		Before	After	Difference	*p*-value
1	81	5.32 (1.34)	6.37 (1.13)	1.05 (1.67)	< 0.0001
2	74	4.22 (1.09)	5.61 (1.04)	1.39 (1.29)	< 0.0001
3	71	4.34 (1.48)	5.11 (1.35)	0.77 (1.70)	< 0.0001
4	69	5.45 (1.26)	6.29 (1.38)	0.84 (1.86)	< 0.0001
5	66	4.27 (1.59)	5.88 (1.84)	1.61 (2.04)	< 0.0001
6	65	4.12 (1.50)	5.68 (1.54)	1.56 (1.84)	< 0.0001
7	63	3.83 (1.20)	5.06 (1.80)	1.23 (1.84)	< 0.0001
Total	489	4.54 (1.47)	5.73 (1.52)	1.19 (1.77)	< 0.0001

Thus, out of 489 responses, most students stated that they would recommend the teaching programme to other students (83.8%).

### Objective 2—To develop students’ confidence in knowledge and skills for common challenges as future doctors

**Fig. 3:**
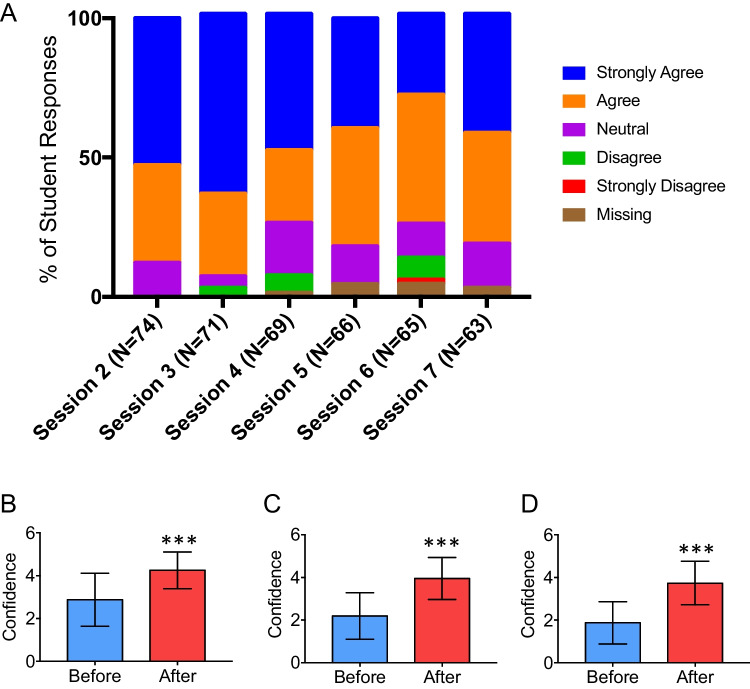
SATMAS prepared students well for common challenges as future doctors. **A**, **B**, **C**, **D** Based on a five-point Likert scale, where 1 is strongly disagree and 5 is strongly agree. **A** Percentage of student responses demonstrating their Likert score of agreement to usefulness of pre-recorded skills videos to help their understanding of the clinical skills. Sixty-three student responses, who completed the whole course, demonstrating their Likert score of agreement, before and after the programme, to **B** assessing a trauma patient through an A-E assessment, **C** suggesting initial investigations and an initial management plan for a trauma patient, **D** managing a trauma patient as a future doctor. *p < 0.05; **p < 0.01; ***p < 0.001

Most students found the pre-recorded skills videos useful (Fig. 3A) and were overall confident in the trauma skills taught during the session (Table 3). Based on the Likert scale, students were more confident in carrying out trauma-related skills (Table 3).

**Table 3:** Student confidence in common trauma skills, stratified by session

Learning objective	Confidence				Students that supported the learning outcome were met, *n* (%)
	Before	After	Improvement	*p*-value	
Session 2: musculoskeletal injury and burns (*N* = 74)					
Application of traction split	1.69	3.76	2.07	< 0.0001	45 (60.8)
Application of pelvic binder	1.97	4.11	2.14	< 0.0001	59 (79.7)
Session 3: airways (*N* = 71)					
Head-tilt chin lift technique	4.06	4.51	0.45	< 0.0001	63 (88.7)
Insertion of nasopharyngeal airway	2.62	4.21	1.59	< 0.0001	60 (84.5)
Insertion of laryngeal mask airway	2.49	4.13	1.64	< 0.0001	56 (78.9)
Insertion of endotracheal tube	2.18	3.87	1.69	< 0.0001	59 (68.1)
Session 4: head and facial injury (*N* = 69)					
Cervical spine immobilisation	2.42	3.41	0.99	< 0.0001	34 (49.2)
Session 5: chest and spinal injury (*N* = 66)					
Needle decompression of chest	2.31	3.98	1.67	< 0.0001	45 (68.2)
Session 6: intra-abdominal injury and shock (*N* = 65)					
Insertion of an intraosseous device	1.69	3.95	2.26	< 0.0001	48 (73.8)
Session 7: pregnant patient with major trauma and non-accidental injury (*N* = 63)					
Suturing	2.19	3.71	1.52	< 0.0001	38 (60.3)

Overall, students that completed the teaching programme reported improvement in their confidence with assessing a trauma patient through an A-E assessment (AD 1.38, *p* < 0.0001), suggesting initial investigations and an initial management plan for a trauma patient (AD 1.78, *p* < 0.001), and thus more confident with managing a trauma patient as a future doctor (AD 1.98, *p* < 0.0001) (Fig. 3B, C, D). As a result of the course, most students stated that their interest in trauma and emergency medicine as a career had also increased (79.4%).

### Objective 3—Determine suitability of pre-recorded online delivery of undergraduate trauma programme.

From 489 student responses, where the majority stated it is better to develop knowledge using online sessions before going straight into practice (81.6%), most supported the use of Moodle as an online platform to deliver the course (78.0%) and the ease of access (83.9%). The most common motivating factors to attend these sessions were ease of access (*n* = 64), lack of travel (*n* = 15), and ability to complete in own time (*n* = 30).

Students who attended all seven sessions were sampled to truly assess suitability of online teaching for undergraduate trauma curricula. After the teaching programme, students reported more preference to teaching via flexible recorded online sessions than face to face (AD 0.78, *p* < 0.001). Similar to individual session feedback responses, students who completed the course stated it is better to develop knowledge using online sessions before going straight into practice (82.5%), and also supported the use of Moodle as an online platform to deliver the course (81.0%) and the ease of access (88.9%).

Where the majority of students who completed the course stated this course was well organised (90.5%), most stated they would recommend this course to future students (88.9%).

## Discussion

Trauma as a specialty for junior doctors focuses on recognising the acutely unwell patient, initiating management and prioritising patient safety. In the UK, junior doctors are often expected to deal with these polytrauma patients. As a result, this leaves one of the most complex challenges in emergency medicine and surgery in the hands of junior doctors. With lack of specific recommendations, and thereby disparities in undergraduate education amongst medical schools, previous studies have highlighted the insufficient medical education and training for junior doctors in trauma [9]. In addition, the transition from medical school to becoming a junior doctor is already difficult and many students worry about their knowledge and competence when first starting [29]. The added responsibilities along with long working hours and further learning impose an additional stress burden. With junior doctors often being the first ones to deal with acutely ill complex trauma patients while under high burdens of stress, our previous research reinforces that they feel poorly prepared to work in trauma, and highlights the need for optimisation of undergraduate medical trauma education [9]. Thus, our programme aimed to provide students with a basis of trauma knowledge to apply once working as a junior doctor. SATMAS was designed to address core concepts such as the primary and secondary survey, suggesting initial investigations, management of different scenarios, and appropriate escalation. Deconstructing the complex approach to trauma into these core concepts enable junior doctors to initiate management, stabilise the patient, and escalate care efficiently and safely to senior team members and other relevant specialties. Thus, such a programme may mitigate the stress of the transition, reduce the burden of extra learning as a doctor, and ultimately benefit patient safety.

Recent studies have shown that OSCEs remain one of biggest sources of exam fear amongst medical students, both pre- and during COVID-19 [28, 30]. Other than practice, one of main reasons for this fear is the lack of thorough knowledge of each skill including the intricate finer details and undergraduate-tailored OSCE-specific techniques. Accordingly, where previous literature has stated use of standardised pre-recorded videos can improve OSCE performance [31], we provided our students with step-by-step tailored pre-recorded demonstrations of core trauma procedures in time-pressured OSCE-style environment. This pre-recorded format allowed students to focus on those intricate details in greater depth with added stop/start function, and re-watch sections that students found difficult, which vary for each student. As a result, students were able to learn and consolidate their knowledge on vital trauma skills, with students who completed the whole course being more confident in encountering a trauma-based scenario in a future OSCE. This was not intended as substitute to in-person teaching but aimed to address the ‘see one’ aspect by providing a strong knowledge base and ensure that students would not practice incorrect technique when the opportunity to practice became available. We publish these results to contribute to limited literature on use of pre-recorded videos for clinical skills and its utility to improve students’ confidence for OSCEs and highlight need for further research in this area to establish it as method.

Competence in trauma and emergency medicine is a vital part of being a junior doctor. Our previous survey of 398 students highlighted a national deficiency in trauma teaching across UK medical schools, with 65.3% of participants not satisfied with current medical school trauma teaching, often using other resources outside of the medial school curriculum [9]. The purpose of this study was to determine whether this novel online trauma course would address NETTS findings to benefit undergraduates. Where our results demonstrate SATMAS improves students’ confidence for their exams, and for common challenges as future doctors (Fig. 3), we propose our course as a useful adjunct to medical schools existing curricula post-COVID-19, rather than disrupting the already busy in-person teaching schedules. With the large discrepancy between the content taught across the country and COVID-19 exacerbating this further [7, 10, 18, 32, 33], we publish this paper to demonstrate the potential of our standardised easily accessible teaching programme to all students from different universities and thereby help to mitigate disparities in learning opportunities post-COVID-19.

Throughout the COVID-19 pandemic, there has been an explosion in the use of online learning [14, 18, 19]. Previous literature has seen the emergence of online platforms to enhance sub-specialty knowledge [34], and there is evidence in other non-trauma specialities that online learning resulted in objectively improved knowledge amongst undergraduates [17]. Additionally, several studies have identified higher participant satisfaction rates with online learning compared to in-person learning [17, 35, 36]. However, while our previous work has demonstrated a significant demand for online trauma teaching, there is little existing research investigating whether online teaching is effective in the context of trauma, particularly at undergraduate level. In the present study, we report both objective improvements in trauma knowledge in MCQs (AD 1.19, *p* < 0.0001) and high satisfaction rates (85.7%) amongst students, thus supporting the utility of online teaching in the context of undergraduate trauma. Additionally, while a limited number of in-person courses and conferences exist [37, 38], these are often restricted to students at specific medical schools, and when open to all, issues such as the cost of travel and timings may be prohibitive. Given the success of online teaching across several other countries and specialties [16, 17, 39], and the easy accessibility, flexibility, and scalability of our programme, we publish our online trauma programme to highlight its potential for development into medical curricula to address the national and potentially global deficiency in trauma teaching at medical schools post-COVID-19 [40].

As with any method of medical education delivery, online learning has its potential limitations including lack of interaction, potential for loss of concentration and loss of tactile learning when teaching clinical skills. Our paper suggests that there is a preference for online sessions rather than face to face and highlights its ongoing role post-COVID; however, a further large-scale study is needed to identify reasons for this preference, and whether this translates into better outcomes. Where we realise most COVID-19 restrictions are no longer in place, this paper conducted during COVID-19 presents the utility of a novel online trauma teaching programme and key areas for further research for its development into medical curricula post-COVID-19. Thus, with utility for both online and in-person methods of medical education delivery, in line with recent studies and other established courses taking on similar approach, e.g. ALS and ATLS [35, 41–43], we recommend a blended approach of prior eLearning (e.g. SATMAS) and practical in-person simulation for undergraduate trauma education post-COVID-19.

There has been debate regarding the use of pre-recorded asynchronous teaching compared to live synchronous teaching. The advantages of pre-recorded teaching include the ability to pause and replay content along with increased flexibility. The advantages of live lectures allow participants to ask questions and allocated times may motivate the students to plan ahead to attend the lectures. Indeed, while some research suggests that there may in fact be equivalence in objective performance outcomes between pre-recorded and live eLearning, subjective outcomes such as student satisfaction are more controversial [44–50]. In the present study, SATMAS prioritised the flexibility of pre-recorded lectures, allowing students to pause and re-visit previous videos for consolidation. With 83.8% of participants in our cohort suggesting that they would recommend this course to their peers, we publish this paper to contribute to existing literature supporting pre-recorded asynchronous teaching and highlight the need for further studies, including RCTs, to fully address role of asynchronous online teaching in medical education.

Trauma as an individual sub-speciality is still not very well represented in the UK [51]. It has been demonstrated in the literature that a lack of speciality exposure may hinder recruitment into the said speciality [52–55]. Given most doctors’ first and only exposure to trauma is in medical school, it is important to maximise student exposure. In addition to SATMAS providing more detailed knowledge of definitive treatment of complex trauma patients, exposure through this formal teaching programme will help to demystify the speciality and allow students to appreciate trauma. This will not only stimulate deeper learning and motivate students for increased online and in-person participation, but inadvertently increase interest in the field and ultimately improve the talent pool of future trauma doctors [52, 56–59]. Where our study showed that after this course, 79.4% of students’ interest in trauma as a career had increased, we propose our SATMAS as a unique way to increase speciality exposure post-COVID-19.

## Limitations

Our pilot study records a small sample size of 489 student responses across seven sessions and 65 of the 90 students who enrolled completed all the sessions. The measures used to ensure suitable test population for pilot may have resulted in relative low participation number: single test centre student recruitment (UoB) and strict deadlines for sign-ups. In addition, where dropouts may represent a source of bias in data collection, our 72.2% completion rate is much higher than is typically reported for voluntary online courses which are often less than 10% [60]. Previous studies have investigated the reasons for dropout in online courses more generally [61], and surveys investigating these factors may not achieve high response rates [62]. However, the use of mandatory feedback forms before and after each individual session allowed us to capture the responses of those who subsequently dropped out and did not complete the entire course. Thus, we were able to gather course-specific data where these students’ felt improvements could be made, for example, adjusting the speed of video recordings. These pre- and post-session feedback forms also allowed the authors to collect sufficient data to demonstrate proof of concept in our pilot, with accurate calculation number of students, unlike other studies [12]. We publish this paper to demonstrate our successful pilot and pave way for further collaboration with organisations to disseminate this programme nationally and larger prospective study into effectiveness and suitability of programme to address the national trauma teaching deficiency.

Our study did not have a control group to compare our online SATMAS to the standard in-person medical teaching. Further, although there was an improvement in students’ knowledge (MCQ) objectively and reported confidence to perform skills subjectively, there was no objective measure used to determine students’ ability to perform clinical skills before and after the session, and long term, so it is difficult to understand how this translates into OSCE performance and clinical practice. The main purpose of SATMAS was to provide teaching when standard medical teaching was not feasible during COVID. Thus, it was not possible to have a control group of standard in person medical teaching, and further it was not possible to construct the ideal OSCE examination before and after the programme. Where the overall aim of this study was not to directly demonstrate superiority of SATMAS over standard medical teaching but to demonstrate proof of concept of our SATMAS online resource, we publish the results of our pilot study to pave way for a large-scale multi-centre randomised control trial comparing SATMAS to a control in-person teaching group, with OSCE assessment before and after the programme, and long-term follow-up, to truly assess the suitability and generalisability of our programme to address the national trauma deficiency.

## Conclusion

To our knowledge, this is the first study investigating the effectiveness of an online undergraduate trauma course in the UK, being first of its kind to pave way for addressing the disparity in trauma teaching in UK medical schools. This pilot study demonstrates that this novel teaching programme is an effective and indispensable COVID-safe resource that prepared undergraduates for their trauma and perioperative exams, laid a strong foundation for implementation of clinical skills required by all doctors, and thereby provided good insight into trauma as a future career. The easily accessible and inexpensive nature of our novel method in trauma teaching not only allow wider outreach to students within same institution but nationally and potentially globally to standardise learning opportunities for tomorrows trauma doctors. Lessons learned from SATMAS’ implementation during the pandemic will help provide novel valid solutions to reduce the ongoing disruption on trauma education and pave way for further research to refine and upscale use of asynchronous online education in trauma, complimentary to in-person teaching post-COVID-19. Contributing to the lack of literature available on undergraduate trauma education, we publish our findings to encourage similar programmes to be adopted by other specialties, to synergistically benefit students, doctors, and ultimately patients.

## Supplementary Information

Below is the link to the electronic supplementary material.

Supplementary file1 (PPTX 56 KB)

Supplementary file2 (DOCX 48 KB)

Supplementary file3 (PPTX 44 KB)

Supplementary file4 (DOCX 25 KB)

Supplementary file5 (PPTX 343 KB)

## Data Availability

The data that support the findings of this study are available on request from the corresponding author (PRK: prakritraj.kumar@nhs.net).
