# Supplementary material for: Student advanced trauma management and skills (SATMAS): a validation study
Source: Eur J Trauma Emerg Surg. 2024 Feb 2;50(4):1407–18. doi: 10.1007/s00068-024-02456-4 (PMC11458672; doi:10.1007/s00068-024-02456-4)
Supplement: Supplementary file 1 — Supplementary file1 (PPTX 56 KB) [file 68_2024_2456_MOESM1_ESM.pptx]

## Slide 1
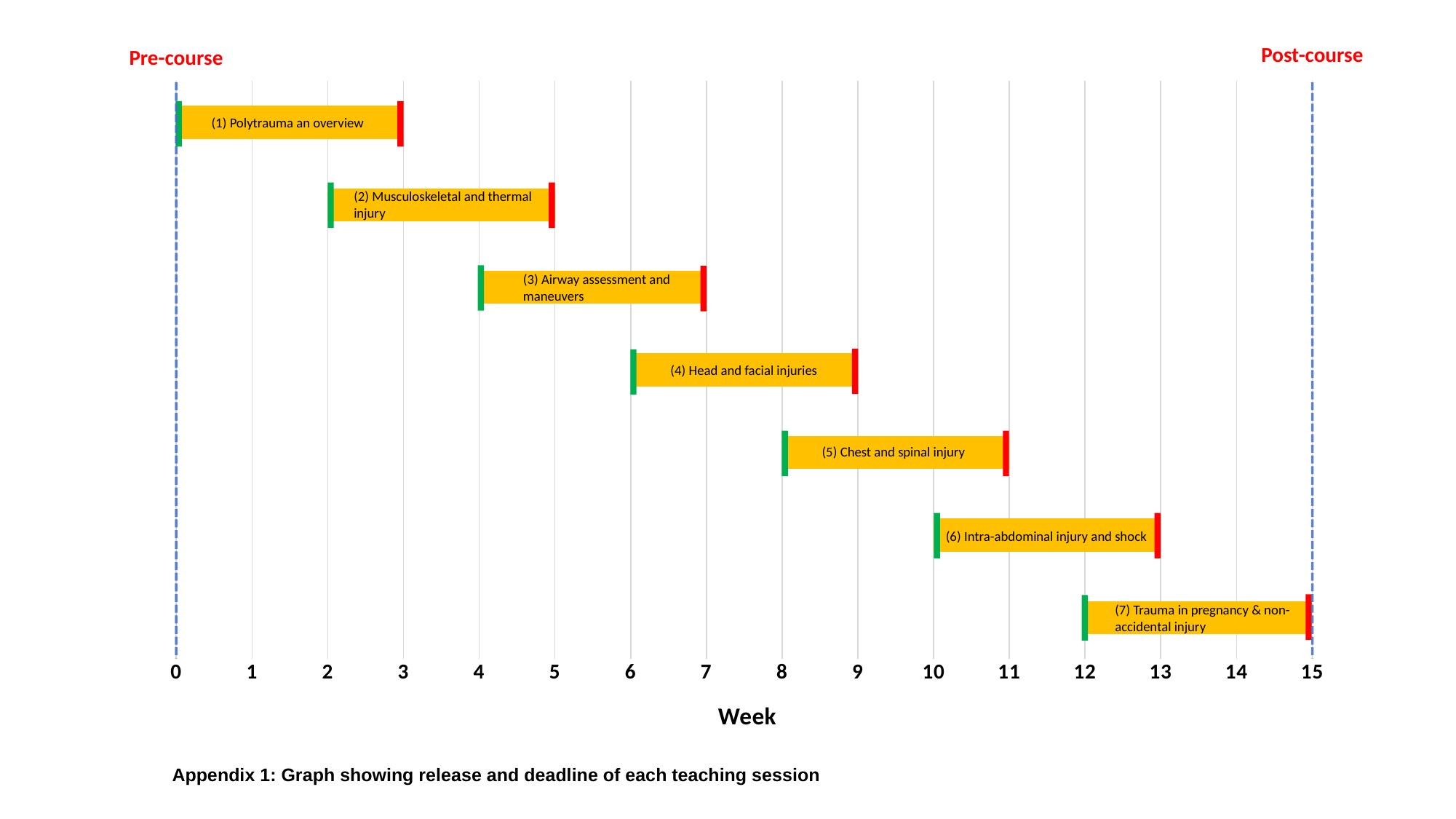

### Chart
| Category | Column1 | Weeks | Duration (weeks) |
|---|---|---|---|(1) Polytrauma an overview
(2) Musculoskeletal and thermal injury
(3) Airway assessment and maneuvers
(4) Head and facial injuries
(5) Chest and spinal injury
(6) Intra-abdominal injury and shock
(7) Trauma in pregnancy & non-accidental injury
Post-course
Pre-course
Appendix 1: Graph showing release and deadline of each teaching session
