# Supplementary material for: Student advanced trauma management and skills (SATMAS): a validation study
Source: Eur J Trauma Emerg Surg. 2024 Feb 2;50(4):1407–18. doi: 10.1007/s00068-024-02456-4 (PMC11458672; doi:10.1007/s00068-024-02456-4)
Supplement: Supplementary file 2 — Supplementary file2 (DOCX 48 KB) [file 68_2024_2456_MOESM2_ESM.docx]

3-6 x Mini lectures

Pre-session Form

Pre-session Test

40 minutes

2

minute

2 minutes

Students sign up via pre-session forms that were disseminated via the University of Birmingham Trauma & Orthopaedics Society 2 weeks prior to session

Pre-session

Session

10 minutes

Pre-recorded skills videos

**Appendix 2:** **Flowchart of the design of the teaching session’s structure**. The process of recruitment and structure of the session with allocated timings.

Post-session Feedback Form

Post-session Test

Post-session

2 minutes

4

minutes
