# Supplementary material for: Student advanced trauma management and skills (SATMAS): a validation study
Source: Eur J Trauma Emerg Surg. 2024 Feb 2;50(4):1407–18. doi: 10.1007/s00068-024-02456-4 (PMC11458672; doi:10.1007/s00068-024-02456-4)
Supplement: Supplementary file 3 — Supplementary file3 (PPTX 44 KB) [file 68_2024_2456_MOESM3_ESM.pptx]

## Slide 1
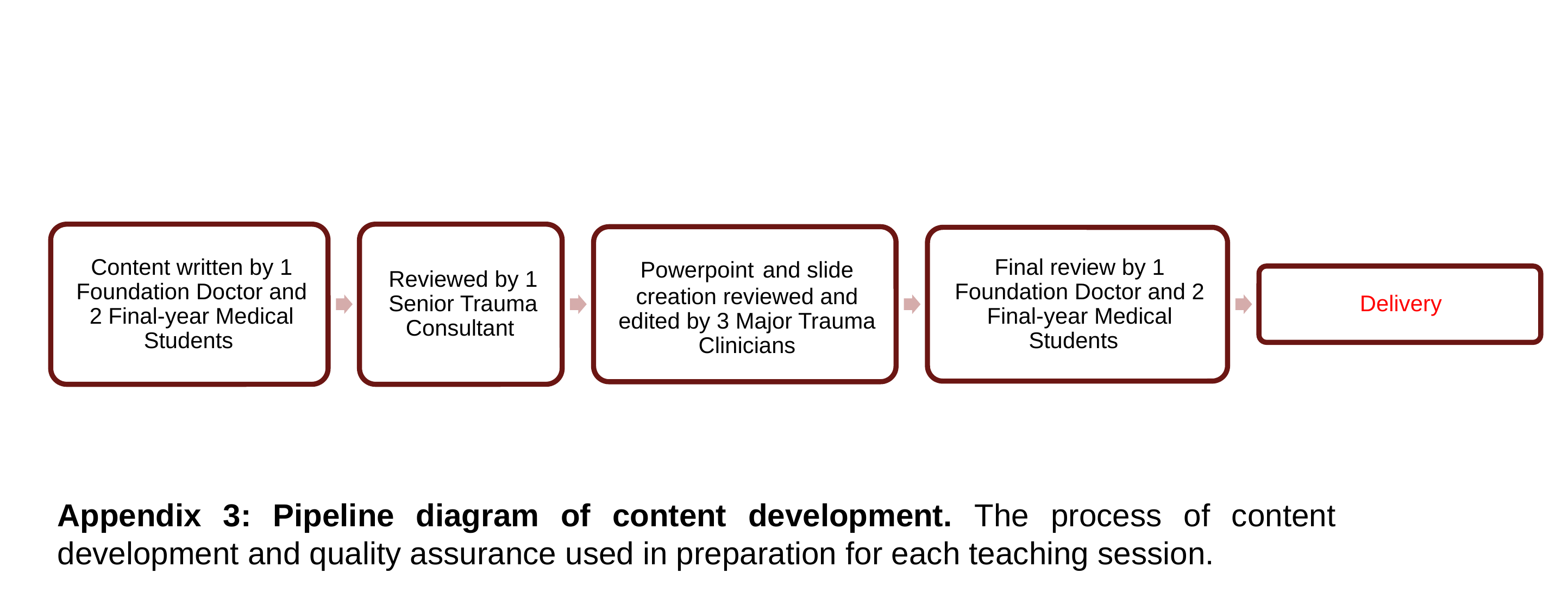

Appendix 3: Pipeline diagram of content development. The process of content development and quality assurance used in preparation for each teaching session.
