# Supplementary material for: Student advanced trauma management and skills (SATMAS): a validation study
Source: Eur J Trauma Emerg Surg. 2024 Feb 2;50(4):1407–18. doi: 10.1007/s00068-024-02456-4 (PMC11458672; doi:10.1007/s00068-024-02456-4)
Supplement: Supplementary file 4 — Supplementary file4 (DOCX 25 KB) [file 68_2024_2456_MOESM4_ESM.docx]

| **Appendix 4A:**  **Pre Teaching Session Feedback Form** | |
| --- | --- |
| **Example - Abdominal Injury and Shock session** | |
| **Part 1:** My overall knowledge of abdominal trauma and shock is (1=Very Poor, 5=Excellent) | **Part 2:** I feel confident in my knowledge of assessing a patient presenting with abdominal injury (1= Strongly disagree, 5= Strongly agree) |
| **Part 3:** I feel confident in my knowledge of assessing a patient presenting with shock (1= Strongly disagree, 5= Strongly agree) | **Part 4:** I am confident with the use of intraosseous devices (1= Strongly disagree, 5= Strongly agree) |

| **Appendix 4B:**  **Post Teaching Session Feedback Form** | |
| --- | --- |
| **Example - Abdominal Injury and Shock session** | |
| **Part 1:** Overall   1. My overall knowledge of abdominal trauma and shock is now (1=Very Poor, 5=Excellent) | **Part 2:** How much to you agree with the following statements regarding the content of the session?  *(1=strongly disagree, 5=strongly agree)*   1. This session met the stated learning objectives 2. Overall, this session was well organised 3. This session covered important topics in the final year medical student curriculum 4. This session had the right amount of information 5. This session was enjoyable and satisfied my expectations 6. This session organised difficult concepts in a clear OSCE style format 7. I now feel confident in my knowledge of assessing a patient presenting with abdominal injury 8. I now feel confident in my knowledge of assessing a patient presenting with shock 9. I am now confident with the use of intraosseous devices |
| **Part 3:** How much do you agree with the following statements regarding the delivery of the session?  *(1=strongly disagree, 5=strongly agree)*   1. The use of videos helped my understanding of the clinical skills 2. Using a trauma specialty SHO/registrar gave me more confidence in the information I was receiving 3. I would prefer to be taught by a final year medical student 4. Using Moodle as a platform was useful to deliver this course 5. Using an online platform enabled me to access course materials remotely and at my own convenience | **Part 4:** How do you rate the following regarding the tutor’s delivery of the session:  *(1=Very Poor, 5=Very good)*   1. Tutor’s Communication and enthusiasm 2. Tutor's level of clinical knowledge 3. Overall effectiveness of the presentation 4. Use of effective teaching methods |
| **Part 5:** Free-text questions   1. Please state 3 positive aspects of the teaching: 2. Please state 3 ways in which this teaching could be improved: | **Part 6:** Further questions   1. What motivated you to attend today's online session? (ease of access, good session organisation, lack of travel) 2. It is better to develop knowledge using online sessions before going straight to practice (1= Strongly disagree, 5= Strongly agree) 3. I would recommend this session to future medical students (1= Strongly disagree, 5= Strongly agree) |

| **Appendix 4C:**  **Pre Teaching Programme Feedback Form** | |
| --- | --- |
| **Part 1:** Demographics   1. What year of study are you in? 2. How did you hear about this course?    1. Not selected    2. Social Media    3. Email    4. Word of mouth 3. Trauma and emergency medicine is an interesting career prospect for me (1=Strongly Disagree, 5= Strongly Agree) | **Part 2:** Current exposure to trauma education   1. I am satisfied with the medical school curriculum for trauma teaching (1=Strongly Disagree, 5= Strongly Agree) 2. I am satisfied with the medical school curriculum for trauma teaching (1=Strongly Disagree, 5= Strongly Agree) 3. I have had experience of trauma themed simulation sessions as part of my degree (1= Strongly Disagree, 5= Strongly Agree) 4. How many hours of bedside trauma teaching have you had as a medical student? 5. How much time have you spent in the Accident and Emergency department as a medical student?    1. Not selected    2. 0-5    3. 6-10    4. 11-15    5. 16-20    6. 20+ |
| **Part 3:** How much do you agree with the following statements regarding the assessment of trauma patients?  *(1=strongly disagree, 5=strongly agree)*   1. I am confident in managing a trauma patient 2. I would be confident in encountering a trauma-based scenario in a future OSCE 3. I am confident with suggesting initial investigations and an initial management plan for a trauma patient 4. I am confident in assessing a trauma patient through an A-E assessment | **Part 4:** How much do you agree with the following statements regarding the delivery of the sessions?  *(1=Strongly Disagree, 5= Strongly Agree)*   1. Teaching via a flexible recorded online session is preferable to face-to-face teaching 2. I would prefer teaching that is accessible remotely and at my own time preference 3. I am more likely to attend an online trauma session rather than face-to-face teaching |

| **Appendix 4D:**  **Post Teaching Programme Feedback Form** | |
| --- | --- |
| **Part 1:** How much do you agree with the following statements about the teaching programme?  *(1= Strongly disagree, 5= Strongly agree)*   1. Overall, this course was well organised 2. My interest in trauma and emergency medicine as a career has increased after this course      1. I would recommend this course to future medical students      1. It is better to develop knowledge using online sessions before going straight to practice 2. Trauma and emergency medicine is now an interesting career prospect for me | **Part 2:** How much do you agree with the following statements regarding the assessment of trauma patients?  *(1=strongly disagree, 5=strongly agree)*   1. I am now confident in managing a trauma patient      1. I would now be confident in encountering a trauma-based scenario in a future OSCE 2. I am now confident with suggesting initial investigations and an initial management plan for a trauma patient 3. I am now confident in assessing a trauma patient through an A-E assessment |
| **Part 3:** How much do you agree with the following statements about the delivery of the teaching programme?  *(1= Strongly disagree, 5= Strongly agree)*   1. Using Moodle as a platform was useful to deliver this course? 2. Using an online platform enabled me to access course materials remotely and at my own convenience 3. Teaching via a flexible recorded online session is preferable to face-to-face teaching 4. What motivated you to attend this online course? (Ease of access, good session organisation, lack of travel) | **Part 4:** How much do you agree with the following statements about the tutors of the teaching programme?  *(1= Strongly disagree, 5= Strongly agree)*   1. Using a trauma specialty SHO/registrar gave me more confidence in the information I was receiving 2. I would prefer to be taught by a final year medical student |
