# Supplementary figures and images for: Student advanced trauma management and skills (SATMAS): a validation study
Source: Eur J Trauma Emerg Surg. 2024 Feb 2;50(4):1407–18. doi: 10.1007/s00068-024-02456-4 (PMC11458672; doi:10.1007/s00068-024-02456-4)

## Slide 1
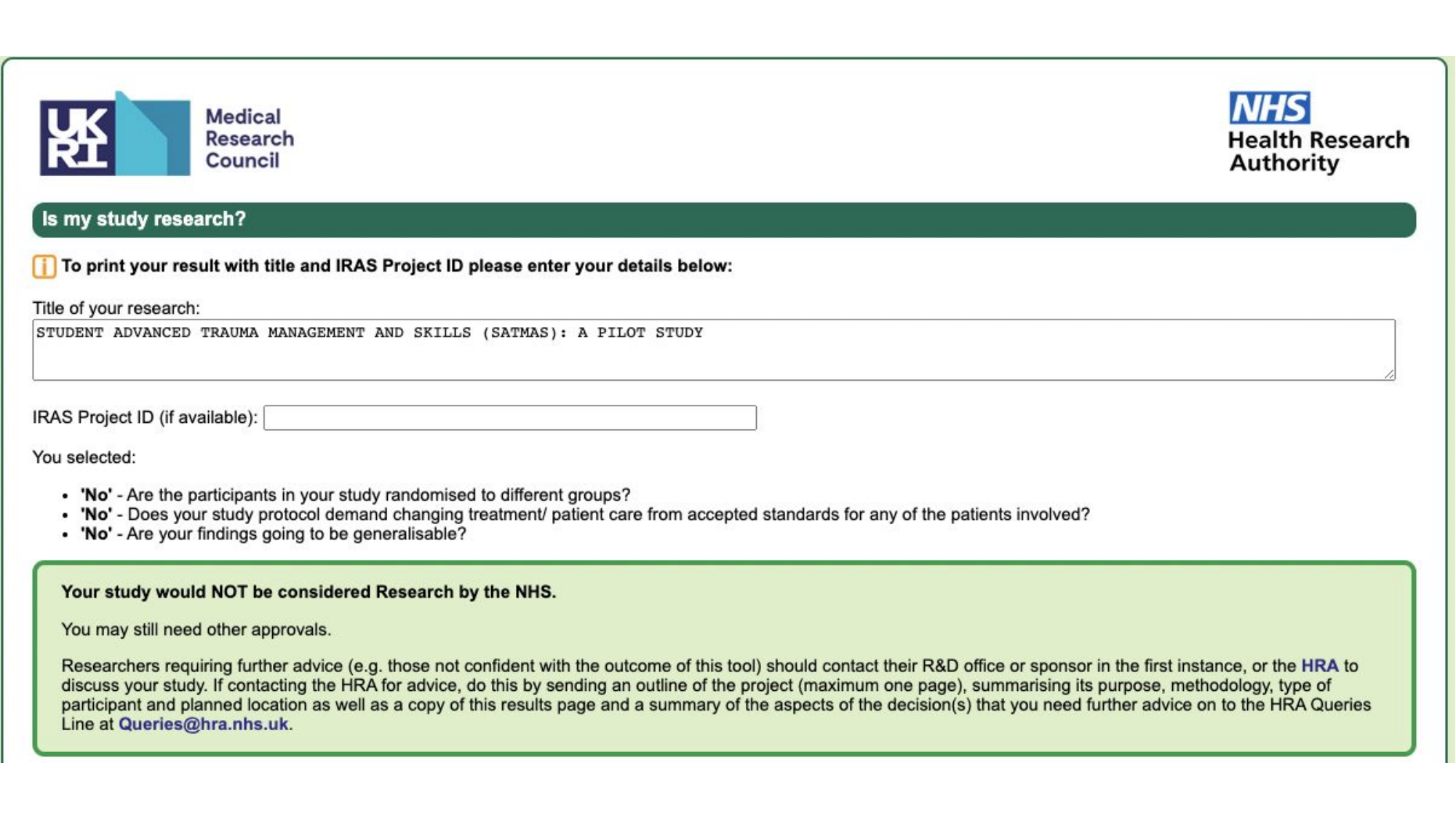

#

Supplement: Supplementary file 5 — Supplementary file5 (PPTX 343 KB) [file 68_2024_2456_MOESM5_ESM.pptx]
